# Preparation and Characterization of Ferrofluid Stabilized with Biocompatible Chitosan and Dextran Sulfate Hybrid Biopolymer as a Potential Magnetic Resonance Imaging (MRI) T2 Contrast Agent

**DOI:** 10.3390/md10112403

**Published:** 2012-10-29

**Authors:** Zei-Tsan Tsai, Fu-Yuan Tsai, Wei-Cheng Yang, Jen-Fei Wang, Chao-Lin Liu, Chia-Rui Shen, Tzu-Chen Yen

**Affiliations:** 1 Molecular Imaging Center, Chang Gung Memorial Hospital, 5 Fu-Hsing Street, Kweishan, Tao-Yuan 33305, Taiwan; Email: zeitsan@ms9.hinet.net (Z.-T.T.); dyy223@yahoo.com.tw (J.-F.W.); 2 General Education Center, Chang Gung University, 259 Wen-Hwa 1st Road, Kweishan, Tao-Yuan 33302, Taiwan; Email: joseph@mail.cgu.edu.tw; 3 Department of Medical Biotechnology and Laboratory Science, College of Medicine, Chang Gung University, 259 Wen-Hwa 1st Road, Kweishan, Tao-Yuan 33302, Taiwan; Email: defoliationtw@gmail.com; 4 Graduate Institute of Biomedical Science, College of Medicine, Chang Gung University, 259 Wen-Hwa 1st Road, Kweishan, Tao-Yuan 33302, Taiwan; 5 Department of Chemical Engineering and Graduate School of Biochemical Engineering, Ming Chi University of Technology, 84 Gung-Juan Road, Taishan, New Taipei 24301, Taiwan; Email: f2402002@ms16.hinet.net

**Keywords:** biocompatible polymer, chitosan, superparamagnetic iron oxide nanoparticle, nanomaterials

## Abstract

Chitosan is the deacetylated form of chitin and used in numerous applications. Because it is a good dispersant for metal and/or oxide nanoparticle synthesis, chitosan and its derivatives have been utilized as coating agents for magnetic nanoparticles synthesis, including superparamagnetic iron oxide nanoparticles (SPIONs). Herein, we demonstrate the water-soluble SPIONs encapsulated with a hybrid polymer composed of polyelectrolyte complexes (PECs) from chitosan, the positively charged polymer, and dextran sulfate, the negatively charged polymer. The as-prepared hybrid ferrofluid, in which iron chloride salts (Fe^3+^ and Fe^2+^) were directly coprecipitated inside the hybrid polymeric matrices, was physic-chemically characterized. Its features include the *z*-average diameter of 114.3 nm, polydispersity index of 0.174, zeta potential of −41.5 mV and iron concentration of 8.44 mg Fe/mL. Moreover, based on the polymer chain persistence lengths, the anionic surface of the nanoparticles as well as the high R2/R1 ratio of 13.5, we depict the morphology of SPIONs as a cluster because chitosan chains are chemisorbed onto the anionic magnetite surfaces by tangling of the dextran sulfate. Finally, the cellular uptake and biocompatibility assays indicate that the hybrid polymer encapsulating the SPIONs exhibited great potential as a magnetic resonance imaging T2 contrast agent for cell tracking.

## 1. Introduction

Magnetic nanoparticles, such as superparamagnetic iron oxide nanoparticles (SPIONs), have been shown to exhibit great potential for use in biomedical applications, such as magnetic separation, magnetic resonance imaging (MRI), targeted drug delivery, and hyperthermia treatment for cancer and have been extensively reviewed [1,2]. In the field of clinical regenerative medicine, iron oxide nanoparticles have been used as a tool for magnetic resonance image tracking of stem cells and have also been recently reviewed [3]. A crucial aspect during the preparation of the SPIONs is to control their size distribution and prevent aggregation [4] by employing macromolecules as coating agents, especially in polysaccharides polymers [5,6,7,8,9]. For *in vivo* MRI contrast agent applications, colloidally monodispersed magnetic nanoparticles, which are known as a ferrofluid, that have a high magnetic relaxivity (r2/r1) ratio and are stable at neutral pH are highly desired [10]. 

**Figure 1:**
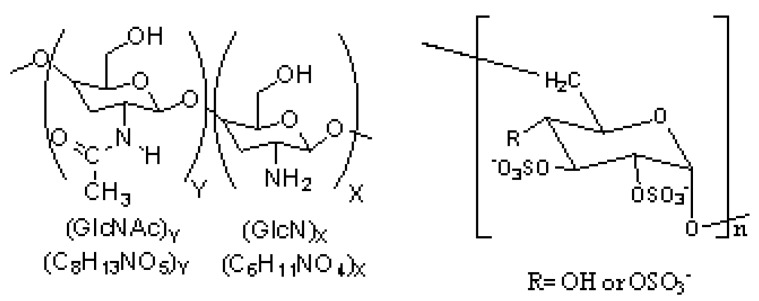
The chemical structures of chitosan (left) and dextran sulfate (right).

Polyelectrolyte complexes (PECs) are formed colloidally in solution by strong electrostatic interactions between charged microdomains of at least two oppositely charged polyelectrolytes. The formation and characterization of positively charged PEC particles, which have submicron sizes, formed by the complexation of chitosan, a weak polybase (Figure 1, left), and dextran sulfate (Figure 1, right), a strong polyacid in the form of a sodium salt, have been studied. Chitosan is a linear copolymer of (1, 4)-linked 2-amino-2-deoxy-β-D-glucan (GlcN) and 2-acetamido-2-deoxy-β-D-glucan (GlcNAc; Figure 1, left) and is primarily generated from the partial *N*-deacetylation of chitin, one of the most abundant renewable polymer in the oceans and is an important source of carbon and nitrogen for marine organisms. The main structural parameter of chitosan is the degree of acetylation (DA); *i.e.*, the molar fraction of acetylated residues in the polymer, which is responsible for both the charge density of the polymer and the balance between the hydrophilic and hydrophobic interactions. In the field of biopolymers, chitosan is well known for its outstanding biological properties, including biodegradability and biocompatibility, which allow for its wide applications [5,9,11,12], including tissue engineering [13,14,15] and drug release [16,17]. Also, the primary amines on chitosan are particularly interesting in metal nanoparticle synthesis due to their interaction with metal ions and metal oxide nanoparticles. Chelation evenly disperses metal oxides throughout the chitosan polymer. Therefore, chitosan (CS) is a good dispersant for iron oxide nanoparticles [9]. Dextran sulfate (DS, Figure 1, right) is primarily known as a substitute for heparin and is a biodegradable negatively charged polymer that is widely used in pharmaceutical applications. DS has a branched chain of anhydroglucose units and contains approximately 17% sulfur, which is equivalent to approximately 2.3 sulfate groups per glucosyl residue [18].

It is important to note that to maintain the colloidal stability of the PECs of chitosan/dextran sulfate, the charge ratio R (n+/n−) should remain less than 1; that is there should be excess dextran sulfate [19,20]. The polymer chain persistence lengths of chitosan and dextran sulfate are quite different, 8.6 nm for chitosan and 1.6 nm for dextran sulfate [20]. The hybrid PEC biopolymer has been reported to encapsulate amphotericin B with zinc sulfate as a crosslinking and hardening agent [18], to protect entrapped insulin from degradation in the gastrointestinal track [21], to controllably release vascular endothelial growth factor [22,23], to encapsulate doxorubicin for assessment of its performance in an osteosarcoma model [24], to entrap curcumin to kill cancer cells [25], and to immobilize a model protein (p24, the capsid protein of the HIV-1 virus) [26]. Optimal incorporation of VEGF was found [23] at a VEGF/DS/CS ratio of 0.12:1:0.33, which resulted in nanoparticle complexes with diameters of 612 nm and a zeta potential of −31 mV. 

The stability of ferrofluids depends on the balance between repulsive and attractive interactions among the magnetic nanoparticles. In addition to thermal motion, steric and electrostatic repulsive interactions act in opposition to van der Waals and dipolar attractive interactions. Therefore, the majority of nanoparticles do not self-assemble into their thermodynamically lowest energy state and require additional energy or external forces (e.g., shear, high energy ultrasound) to direct them into particular structures or assemblies [27]. Subsequently, these nanoparticles require a period of standing to reach their thermodynamically lowest energy state. We have developed an in situ preparation method for high-relaxivity iron oxide nanoparticles that involves coating the particles with γ-ray irradiated solid chitosan [9] or *N*-[(2-hydroxy-3-trimethylammonium) propyl] chitosan chloride [28,29], and we demonstrated that the ferrofluids we prepared could be used as an MRI contrast agent for cell tracking [30,31]. The main objective of the present study was to prepare hybrid SPION ferrofluids stabilized with biocompatible chitosan and dextran sulfate polymers and to study the potential of these ferrofluids for use as MRI T2 contrast agents for cell tracking. The physicochemical properties of hybrid SPION ferrofluids including *z*-average diameter, zeta potential, iron concentration and proton relaxivity were analyzed. In addition, the ferrofluids were imaged using transmission electron microscopy (TEM). Finally, the cellular uptake and biocompatibility were also evaluated.

## 2. Results and Discussion

### 2.1. Synthesis of the Hybrid Ferrofluid (Hybrid SPIONs)

Chitosan (CS) and dextran sulfate (DS) are positively and negatively charged polysaccharides, respectively. DS is made up of repeating units of glucose sulfate, and CS is composed of repeating units of glucosamine/*N*-acetyl-glucosamine. Ionic interactions between CS and DS produce the PECs. The final particle size and charge of the PECs are determined by the molecular weight of each of the polymers and the weight ratio between them [23]. The formation of colloidal PECs from chitosan and excess dextran sulfate has been proposed by Delair [19] and Zhang [23]; in these PECs, the two types of polymer chains were tangled together with the dextran sulfate chains on the outside. This has led us to set the weight ratio for chitosan to dextran sulfate to 1:1.5 to obtain colloidal PECs in 1 M acetic acid [19,23]. After centrifugation, the supernatant was collected and diluted 200-fold with deionized water. Then, the *z*-average diameter, polydispersity index and zeta potential of the PECs were measured. These values were 150.3 nm, 0.181, and −32.2 mV, respectively, which are in good agreement with previously reported values [23]. However, the PECs collapsed as precipitation occurred in PBS and cell culture medium, such as RPMI medium 1640 [23]. 

To synthesize the hybrid ferrofluid (hybrid SPIONs) stabilized with biocompatible chitosan and dextran sulfate with a weight ratio of 1:1.5, the so-called in situ coating method was adapted from previously published protocols [18,28,29]. The iron chloride salts were directly coprecipitated as SPIONs inside the hybrid polymeric matrices upon addition of ammonia water in an acetic acid solution. The chitosan was previously gamma-ray irradiated to render it soluble. 

### 2.2. Physicochemical Features of the Hybrid Ferrofluid

**Figure 2:**
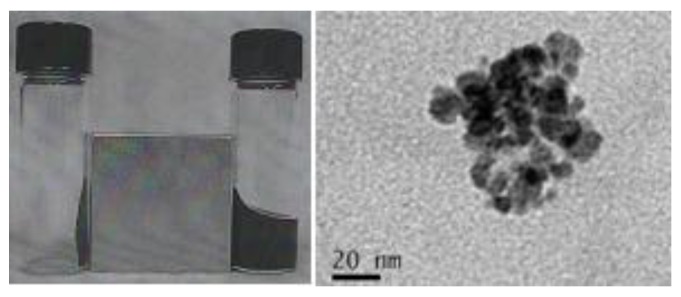
Left: photograph of the hybrid ferrofluid (right) and its dried sediment (left) under an external magnet. Right: Transmission electron microscopy (TEM) morphology analysis of the hybrid ferrofluid.

After obtaining the as-prepared hybrid ferrofluid, we characterized its physicochemical properties. The left panel of Figure 2 shows the images of the hybrid ferrofluid (right) and its dried sediment (left) under an external magnet. The particles remained dispersed in the fluid even in the presence of the external field, indicating that a stable colloidal solution had formed, and the particles retained their superparamagnetic nature and high magnetite content. The right panel of Figure 2 shows the TEM morphology of the hybrid ferrofluid and illustrates the structural characteristics of the nearly monodisperse magnetite nanoparticles synthesized in the hybrid biopolymer matrix. 

**Figure 3:**
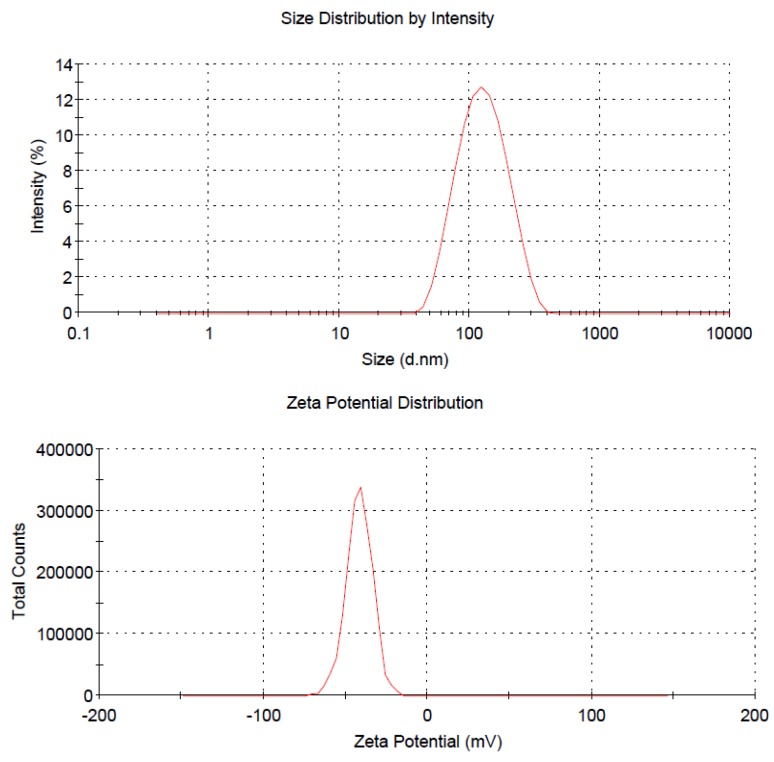
Upper: size distribution of the hybrid ferrofluid. Lower: zeta potential distribution of the hybrid ferrofluid.

Next, the ferrofluid was characterized by measuring its *z*-average diameter and zeta potential with the laser light-scattering apparatus in water. The average size was 114.3 nm in diameter, and the particles carried a negative zeta potential of −41.5 mV in deionized water. Figure 3 shows the size distribution (Figure 3, upper) and the zeta potential (Figure 3, lower) from one representative experiment in which a 200-fold dilution of the hybrid ferrofluid was prepared using deionized water. These results indicate that the as-prepared ferrofluid contains a fairly unimodal distribution of monodisperse clusters. Moreover, the polydispersity index (PDI) and the iron content were also determined and found to be 0.174 and 8.44 mg/mL, respectively, for the as-prepared hybrid ferrofluid. However, the numerical magnitudes for both the hybrid ferrofluid and the colloidal PECs were somewhat affected by the dilution in water and are tabulated in Table 1. In the cumulants analysis, a single particle size is assumed and a single exponential fit is applied to the autocorrelation function. The 1st Cumulant or moment (a1) is used to calculate the intensity weighted Z average mean size and the second moment (a2) is used to calculate a parameter defined as the polydispersity index (PDI). In other words, the PDI value in the light scattering area is related to the colloidal stability and should be less than 0.3. If the PDI value of a solution is equal and/or greater than 1, then the solution may occur visual precipitation. In Table 1, the PDI values are ranging from 0.120 to 0.181 reflecting good colloidal properties. Similar to the PECs, the as-prepared hybrid ferrofluid particles collapsed as precipitation occurred in PBS and cell culture medium, such as RPMI-1640. 

**Table 1:** The *z*-average diameter, polydispersity index, and zeta potential for both the as-prepared hybrid ferrofluid and the colloidal polyelectrolyte complexes (PECs) diluted in H_2_O.

Dilution	*z*-average diameter (nm)		polydispersity index		zeta potential (mV)	
	Ferrofluid	PECs	Ferrofluid	PECs	Ferrofluid	PECs
Stock ^a^	194	133.3	0.173	0.150	−22.7	−36.8
2×	152.9	134.4	0.163	0.134	−33.4	−38.3
5×	133.7	140.4	0.179	0.120	−39.4	−38.7
10×	124.9	147.3	0.176	0.123	−40.2	−32.7
200×	114.3	150.3	0.174	0.181	−41.5	−32.2

^a^ After centrifugation at 6000 rpm for 20 min, the supernatant was used as the stock solution for both the hybrid ferrofluid and the colloidal PEC. The concentration of the hybrid ferrofluid stock solution was 8.44 mg Fe/mL.

### 2.3. Relaxometry of the Hybrid Ferrofluid

**Figure 4:**
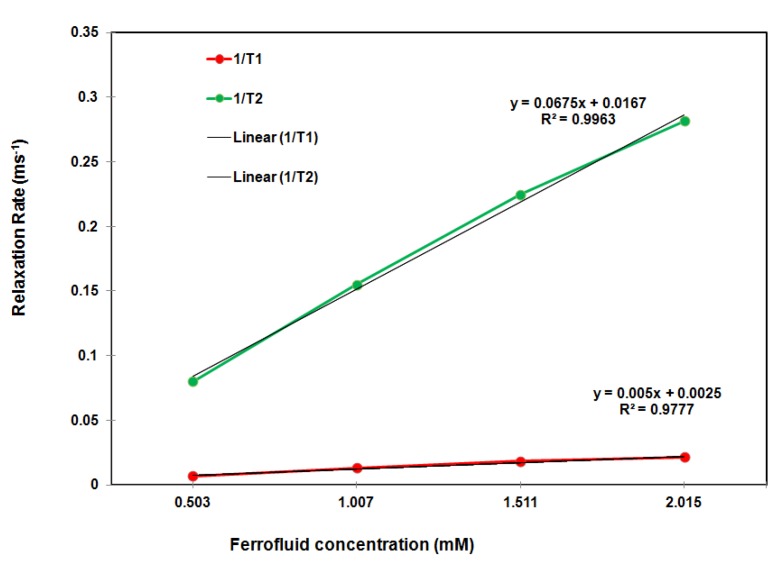
T1/T2 relaxation rate (ms^−1^) as a function of the Fe concentration (mM) of the hybrid ferrofluid in water measured using the 0.47 T NMR spectrometer.

A potential MRI contrast agent is usually evaluated based on its relaxivities, R1 and R2, which are the rates of proton relaxation. To assess the relaxivities of the as-prepared hybrid ferrofluid, a 0.47 T Bruker Minispec mq-20 20 MHz relaxation time analyzer was used for the T1 and T2 measurements. Then, the relaxivities, R1 and R2 (mM^−1^ s^−1^), were calculated from a linear least squares regression analysis of the relaxation rate (ms^−1^) as a function of the sample concentration (mM Fe) with an R2 greater than 0.9988. In general, the higher the R2/R1 ratio, the better the contrast efficacy for a T2 contrast agent. As shown in Figure 4, R2 = 67.5 mM^−1^ s^−1^, R1 = 5.0 mM^−1^ s^−1^ and R2/R1 = 13.5 (0.47 T NMR analyzer), which indicates that the hybrid ferrofluid shows promise as a potential MRI T2 contrast agent. Given the polymer chain persistence lengths of 8.6 nm and 1.6 nm for chitosan and dextran sulfate, respectively, the anionic surface of the iron oxide nanoparticles and the high R2/R1 ratio of 13.5, the morphology of the SPIONs in the PECs could be depicted as a cluster in which the chitosan chains are chemisorbed onto the anionic magnetite surfaces [9,10] and are entangles by the dextran sulfate polymer chains. 

### 2.4. Biocompatibility and Cellular Uptake Analysis

**Figure 5:**
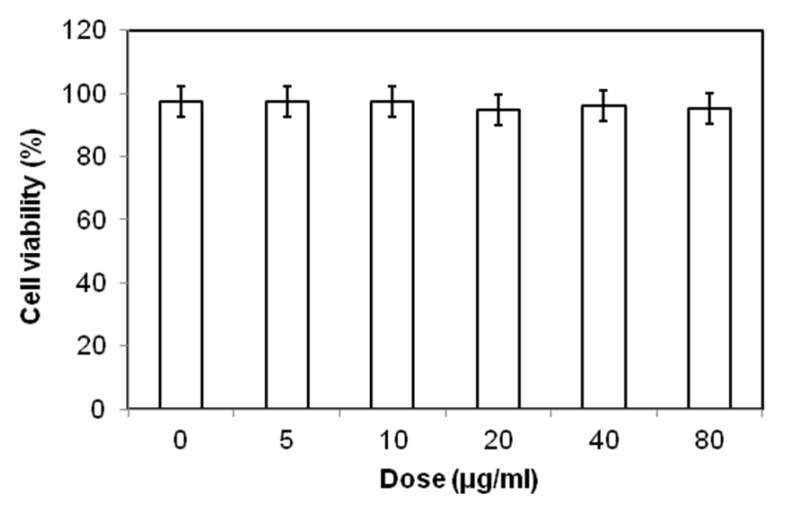
Biocompatibility analysis of the hybrid ferrofluid on cell survival.

An ideal cell-tracking agent must be biocompatible and easily taken up by cells. Therefore, the toxicity profile of the as-prepared ferrofluid was studied *in vitro*. The representative cytotoxic effect of the ferrofluid on cell survival is shown in Figure 5. The 3T3 cells were loaded with different concentrations of the hybrid ferrofluid, and the effect of the SPION loading on cell survival was examined using propidium iodide (PI) staining. The cell viability assays revealed no evidence of toxicity across a 16-fold dose range (5–80 μg/mL) of the hybrid SPIONs. The trypan blue exclusion experiments confirmed these results (data not shown). In our previous studies [28,29], we performed similar experiments and assessed the long-term effects of SPION loading at 48, 72 and 96 h. If such materials were harmful, we observed direct and immediate toxicity. 

**Figure 6:**
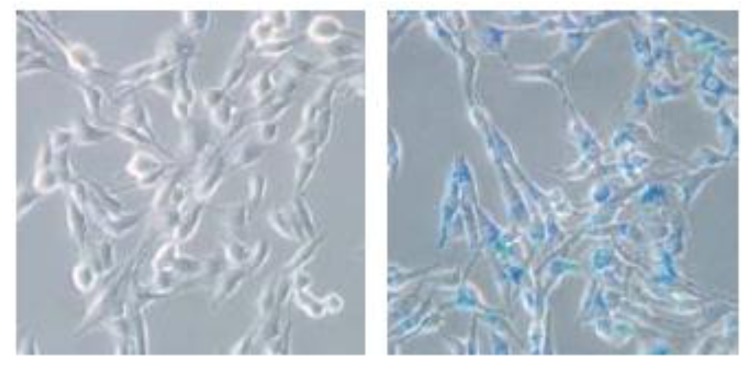
A typical Prussian blue stain (100×) for BALB/c 3T3 fibroblasts before (left) and after (right) uptake of the hybrid ferrofluid.

Finally, the most important factor is the cellular uptake efficiency of these magnetic particles. The uptake of representative SPIONs is shown in Figure 6, with a typical Prussian blue staining for the fibroblast cell line (3T3) after uptake of the hybrid ferrofluid. This assay demonstrated that the SPIONs were found inside the cells after an overnight incubation, which indicated the effective cell internalization of the nanoparticles. 

## 3. Experimental Section

### 3.1. Materials

Iron(III) chloride hexahydrate (FeCl_3_·6H_2_O) and D-mannitol were obtained from Riedel-de Haen (Germany). Iron(II) chloride tetrahydrate (FeCl_2_·4H_2_O) was obtained from Showa (Japan). Ammonium hydroxide solution (25%) was obtained from Fluka (Germany). Chitosan (medium Mw, 190–310 kDa, 93.4% deacetylated, Aldrich, batch No. 08028CD) and dextran sulfate sodium salt from *Leuconostoc* spp. (Mw = 100 kDa, Fluka) were used as received without further purification. Solid chitosan was subjected to Co-60 g-ray irradiation with a dose of 300 kGy prior to use. After irradiation, the molecular weight of chitosan was approximately 16.2–16.8 kDa [32]. Deionized water was purged with nitrogen gas for 30 min prior to use.

### 3.2. Preparation of the Hybrid SPION Ferrofluid

Approximately 0.31 g of chitosan (gamma-ray irradiated) and 0.465 g of dextran sulfate sodium salt were dissolved in 100 mL of 0.5% (v/v) aqueous acetic acid. Then, 1.00 g of FeCl_3_·6H_2_O and 0.45 g of FeCl_2_·4H_2_O were added to the polymer solution to afford a pale brown solution. Then, 15 mL of 29% ammonia water was rapidly added to the brown solution under sonication at 50 °C, and sonication was continued for 40 min. The black precipitate was isolated with a magnet and decanted or centrifuged at 5000 rpm for 5 min and washed with 95% ethanol at least three times until no AgCl cloud formed when silver nitrate solution was added. The washed black precipitate yielded the hybrid ferrofluid when it was dispersed in 40 mL of water, and the pH was adjusted to approximately 7.0 with lactic acid and then adding mannitol. After centrifugation at 4000 rpm for 20 min [9,33], the supernatant was collected, and its physicochemical properties were determined. 

### 3.3. Characterization of the Hybrid SPION Ferrofluid

The particles of the hybrid SPION ferrofluid were imaged using a 200-keV JEOL-2000 FXII transmission electron microscope to determine the nanoparticle morphology. The stability and structure relaxation time of the hybrid ferrofluid were determined via dynamic light scattering (DSL) using a Zetasizer Nano ZS instrument (Malvern Instruments Ltd., UK) that allows specific measurement of the *z*-average diameter, which is defined as the intensity-weighted average hydrodynamic diameter of the particles being measured, and the zeta potential. The autocorrelation function was fitted using exponential fitting software to extract the diffusion coefficient. The Stokes-Einstein equation was used to convert the diffusion coefficient into a hydrodynamic diameter. The stock sample and various samples diluted in water (2×, 5×, 10×, and 200×) were analyzed. An aliquot of 30 μL of the sample was added to 1.5 mL of deionized water and evenly mixed. A 0.5-mL aliquot of the sample was passed through a 0.45-μm Millipore filter and then transferred to the cells and analyzed. The colloidal PECs of chitosan and dextran sulfate were prepared using the same weight ratio. Their *z*-average diameter and the zeta potential of the supernatant (pH = 2.66) after centrifugation at 6000 rpm for 20 min were determined using the same method used for the hybrid SPION ferrofluid.

The iron concentration of the hybrid ferrofluid was determined colorimetrically using *O*-phenanthroline as a chelator; the absorbance at 510 nm was measured as described in a previously published protocol [9]. 

A 0.47 T Bruker Minispec mq-20 20 MHz relaxation time analyzer was used for T1 and T2 measurements. First, 10 μL to 40 μL of each sample was diluted with deionized water to adjust the concentration to 0.503–2.015 mM Fe, and then the diluted sample was placed in a 10-mm sample tube and allowed to equilibrate to 37 °C in a water bath. All of the measurements were made at 37 °C using a temperature-controlled probe cavity with an external water bath. The T1 curve was obtained using the instrument’s inversion-recovery pulse sequence (t1_ir_mb) with a single scan, a recycle delay of 1cycle, a first duration of 1 ms, a last duration of 1000 ms, 20 data points, and monoexponential curve fitting. T2 curves were obtained using the instrument’s Carr-Purcell-Meiboom-Gill (CPMG) pulse sequence (t2_cp_mb) with a recycle delay of 1 s, pulse separation of 0.5 ms, 100 data points, dummy echoes of 0 and monoexponential curve fitting. The relaxivities, R1 and R2 (mM^−1^ s^−1^), were calculated from a linear least squares regression analysis of the relaxation rate (ms^−1^) as a function of the sample concentration (mM Fe) with R2 greater than 0.9963. 

### 3.4. Cell Survival

BALB/c 3T3 fibroblast cells and other cell lines were used to evaluate the biocompatibility of the hybrid ferrofluid in cell survival experiments. Briefly, the cells were seeded into culture plates and exposed to different concentrations of SPIONs for 24 h. Cells loaded with SPIONs were washed with phosphate-buffered saline (PBS) and trypsinized. Cell survival was evaluated by propidium iodide (PI) exclusion according to previously published protocols [28,29]. Alternatively, trypan blue exclusion analysis using a hemocytometer and microscope was also utilized to confirm the results. 

### 3.5. Cellular Uptake

BALB/c 3T3 fibroblast cells and other cell lines were used in cellular uptake experiments. Briefly, the cells were seeded into culture plates and exposed to different concentrations of SPIONs for 24 h. After a washing step to remove the excess iron particles, the cells were then fixed in 4% (v/v) formaldehyde solution for 30 min. After fixation, the cells were stained for the presence of intracellular iron with freshly prepared potassium ferrocyanate solution (i.e., a mixture of equal volumes of 4 wt% potassium ferrocyanate and 4 vol% hydrochloric acid) for 30 min. After washing with distilled water, the cells were scored using a microscope. Any cells that contained blue or brown particles were considered positive [28,29].

## 4. Conclusions

SPIONs encapsulated by hybrid polymers composed of chitosan and dextran sulfate appear to be biocompatible and exhibited good cellular uptake and magnetic relaxivity. These characteristics of the as-prepared hybrid SPIONs support their use as a potential MR contrast agent for cell tracking. 
